# Molecular cloning and expression profiling of a chalcone synthase gene from hairy root cultures of *Scutellaria viscidula* Bunge

**DOI:** 10.1590/S1415-47572010005000031

**Published:** 2010-06-01

**Authors:** Wei Lei, Shao-Hu Tang, Ke-Ming Luo, Min Sun

**Affiliations:** Key Laboratory of Eco-environments in Three Gorges Reservoir Region of Ministry of Education, School of Life Science, Southwest University, ChongqingChina

**Keywords:** chalcone synthase gene, methyl jasmonate, molecular cloning, *Scutellaria viscidula* Bunge

## Abstract

A cDNA encoding chalcone synthase (CHS), the key enzyme in flavonoid biosynthesis, was isolated from hairy root cultures of *Scutellaria viscidula* Bunge by rapid amplification of cDNA ends (RACE). The full-length cDNA of *S. viscidula* CHS, designated as *Svchs* (GenBank accession no. EU386767), was 1649 bp with a 1170 bp open reading frame (ORF) that corresponded to a deduced protein of 390 amino acid residues, a calculated molecular mass of 42.56 kDa and a theoretical isoelectric point (pI) of 5.79. Multiple sequence alignments showed that SvCHS shared high homology with CHS from other plants. Functional analysis *in silico* indicated that SvCHS was a hydrophilic protein most likely associated with intermediate metabolism. The active sites of the malonyl-CoA binding motif, coumaroyl pocket and cyclization pocket in CHS of *Medicago sativa* were also found in SvCHS. Molecular modeling indicated that the secondary structure of SvCHS contained mainly α-helixes and random coils. Phylogenetic analysis showed that SvCHS was most closely related to CHS from *Scutellaria baicalensis*. In agreement with its function as an elicitor-responsive gene, the expression of *Svchs* was induced and coordinated by methyl jasmonate. To our knowledge, this is the first report to describe the isolation and expression of a gene from *S. viscidula*.

## Introduction

**Figure 1:**
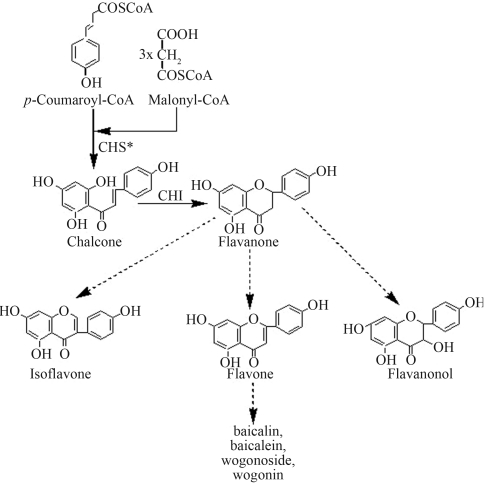
Flavonoid biosynthetic pathway in *Scutellaria* cells.

Flavonoids are a large group of widespread plant secondary metabolites involved in numerous biological processes such as protection against UV light, flower coloration, defense against pathogens and pollen development (Winkel, 2002). Chalcone synthase (CHS, EC 2.3.1.74) catalyzes the first committed step of the flavonoid biosynthesis pathway in which three acetate residues from malonyl-CoA and *p*-coumaroyl-CoA are condensed to form naringenin chalcone (Stefan and Axel, 2005) (Figure 1). The *chs* gene has been extensively studied in a variety of plants, including *Oryza sativa*, *Zea mays* and *Medicago sativa*.

Plants of the genus *Scutellaria* contain several medicinally important flavonoids, including baicalin, baicalein, wogonoside and wogonin, that are extracted mainly from the roots of representative species such as *Scutellaria baicalensis* Georgi and *Scutellaria viscidula* Bunge. The medicinal properties of these root extracts are well-known in traditional Chinese medicine and are widely used to treat inflammatory and bacterial diseases in oriental countries (Yamamoto, 1991). Recent studies have shown that *Scutellaria* flavonoids have antiviral activity against human immune-deficiency virus (HIV-1) and T-cell leukemia virus (HTLV-1) (Kovács *et al.*, 2004), inhibit the growth of breast, hepatocellular, pancreatic, prostatic, urothelial and colon cancer cells *in vitro* (So *et al.*, 1997; Ikemoto *et al.*, 2000; Ye *et al.*, 2002), and scavenge free-radicals (Shieh *et al.*, 2000). However, little information is available on the enzymes involved in flavonoid biosynthesis in *Scutellaria* species.

Methyl jasmonate (MeJA), an endogenous phytohormone, is an important signaling agent and potent elicitor (Yukihito *et al.*, 1996) involved in a range of physiological responses with key roles in plant development (Creelman and Mullet, 1997). Although the mechanism of interaction of MeJA with specific receptors in plant cells remains unclear, there has been increasing interest in the pathways involved in MeJA-mediated activation and regulation of genes coding for enzymes involved in plant secondary metabolism (Heidrun *et al.*, 1992). For example, treatment with MeJA stimulates *pal* (phenylalanine ammonia lyase), *chs* (chalcone synthase), *sts* (stilbene synthase) and *ubgat* (UDP-glucose:flavonoid 7-O-glucosyltransferase) expression in *Vitis vinifera* L. (Assia *et al.*, 2008), and enhances the antioxidant activity and flavonid content in blackberries (Wang *et al.*, 2008b).

*Scutellaria viscidula* has a wide distribution in the mountains of Hebei, Shanxi and other provinces in northern China. This species is frequently used as a substitute for the skullcap *S. baicalensis*, for which the demand exceeds supply, primarily because of dwindling natural populations and the difficulty in breeding this species on a commercial scale. The content of medically important flavonoids in *S. viscidula* has been reported to be higher than in *S. baicalensis* (Wang *et al.*, 2007). In this report, we provide the first description of the molecular cloning of a *chs* gene from *S. viscidula* by rapid amplification of cDNA ends (RACE). The expression profile of *svchs* following induction by MeJA was also investigated. These results broaden our understanding of the structure of the *chs* gene and the molecular mechanisms involved in flavonoid biosynthesis.

## Materials and Methods

###  Plant material and treatment with elicitor

Hairy roots of *S. viscidula* Bunge were obtained from seedlings and cultivated in MS_0_ medium (Murashige and Skoog, 1962), as previously described (Wang *et al.*, 2008a). Optimal hairy root lines were subsequently grown in the liquid 1/2MS_0_ medium on a rotary shaker (110 rpm) at 25 ± 1 °C.

MeJA was added to liquid 1/2MS_0_ medium (final concentration: 200 μmol/L) and 150 mL of this solution was placed in 250 mL conical flasks followed by the inoculation of ~1 g of fresh hairy roots (3 cm in length). The roots were grown in suspension and after 6, 12, 24, 48 and 72 h samples were obtained to assess *chs* gene expression, as described below. Appropriate blanks were run in parallel.

###  Isolation of total RNA

Total RNA was extracted from *S. viscidula* shoots and from blank hairy roots with TRIzol reagent according to the manufacturer's instructions (Tiangen, Beijing, China). The quality of RNA were determined by agarose gel electrophoresis and the amount of RNA was quantified spectrophotometrically (DU-640, Beckman, USA). The RNA was stored at -70 °C until used.

###  Cloning of full-length *Svchs* cDNA by RACE

Single-strand cDNA was synthesized using TaKaRa RNA PCR kit ver. 3.0 (TaKaRa, Dalian, China) according to the manufacturer's instructions. After RNaseH treatment, the single-stranded cDNA mixture was used as a template for the polymerase chain reaction (PCR). Two oligonucleotide primers (dfchs and drchs, Table S1) were designed based on highly conserved regions in known *chs* genes from other plant specieses. The PCR reaction was run using the following protocol: initial denaturing at 94 °C for 3 min, followed by 34 cycles of denaturing for 30 s at 94 °C, annealing for 30 s at 55 °C and extension for 1 min at 72 °C, with a final extension for 10 min at 72 °C. After electrophoresis the PCR products were recovered from the agarose gels by using a DNA gel extraction kit (Watsonbiot, Shanghai, China), after which the fragments were ligated into the vector pMD18-T (TaKaRa) and introduced into competent *Escherichia coli* strain DH5α cells. Recombinant plasmids recovered from positive colonies were sequenced to identify the core fragment.

A SMART RACE cDNA amplification kit (Clontech, USA) was used to isolate *Svchs* 3'- and 5'-end cDNA. The first-stranded 3'-RACE-ready and 5'-RACE-ready cDNA samples from *S. viscidula* were prepared according to the manufacturer's protocol. A universal primer A mix (UPM, provided in the kit), the 3'-gene-specific primer svchs3-1 (Table S1) and 3'-ready cDNA were used for the first round of 3' RACE. For the nested PCR amplification of 3'-RACE, svchs3-2 (Table S1) and nested universal primer A (NUP, provided in the kit) were used.

The 5'-ready cDNA was synthesized with the 5'-CDS primer A and SMART II A oligonucleotide provided in the kit. Two 5'-gene-specific primers, svchs5-1 and svhcs5-2 (Table S1), were designed for 5'-RACE. The first round of PCR amplification was done with primers svchs5-1 and UPM under the following conditions: 1 min at 94 °C, 30 cycles of 30 s at 94 °C, 30 s at 58 °C, 2 min at 72 °C, and finally 10 min at 72 °C. Subsequently, the PCR products were used as templates for nested PCR amplification with the primers svchs5-2 and NUP. The PCR amplification was done using the following conditions: 3 min at 94 °C followed by 34 cycles of 30 s at 94 °C, 30 s at 55 °C and 2 min at 72 °C, and a final 10 min at 72 °C. The PCR products were purified, subcloned into the vector pMD18-T and transformed into *E.coli* strain DH5α followed by sequencing.

After aligning and assembling the sequences of the core fragment and 3' and 5' RACE ends on ContigExpress (Vector NTI Suite 6.0), the full-length cDNA sequence of *svchs* was obtained *in silico* and was used to design a pair of specific primers (fsvchs and rsvchs; Table S1). The full-length *svchs* cDNA was subsequently amplified by RT-PCR with the specific primer pair using the following conditions: 3 min at 94 °C, 34 cycles of 30 s at 94 °C, 30 s at 55 °C and 2 min at 72 °C, and a final extension for 10 min at 72 °C min. The PCR products were purified and cloned into the vector pMD18-T followed by sequencing. PCR amplification and sequencing were repeated three times to confirm the results.

###  Bioinformatic analysis

The nucleic acid sequence of the *svchs* gene (GenBank accession no. EU386767) and corresponding amino acid sequence of the protein (SvCHS) were calculated and analyzed with bioinformatics computer tools. Comparative bioinformatic analysis was done online at the NCBI and Expasy websites. The open reading frame (ORF) was predicted by ORF Finder. Multiple sequence alignments of the amino acid sequences of SvCHS and CHSs from other plant specieses were done with Vector NTI 8.0 using default parameters (Lei *et al.*, 2009). Subcellular location was predicted with the TargetP 1.1 server (Kristin and Siegfried, 2004). Cellular function, transmembrane helices and hydrophobicity of the protein were predicted with the ProtFun 2.2 server (Jensen *et al.*, 2002, 2003), TMHMM server v.2.0 (Ikeda *et al.*, 2002) and ProtScale (Kyte and Doolittle, 1982), respectively. Protein motifs were identified with ScanProsite (Combet *et al.*, 2000). SvCHS and other plant CHS were aligned with ClustalX (Thompson *et al.*, 1997) and subsequently a phylogenetic tree was constructed by the Neighbor-Joining method with 1000 replicates; the reliability of each node was established based on bootstrap calculations using MEGA3 software (Kumar *et al.*, 2001). Homology-based three-dimensional (3D) structural modeling of the SvCHS protein was done with Swiss-Modeling (Arnold *et al.*, 2006) and WebLab ViewerLite 4.2 was used to display the 3D structure.

###  Expression profile analysis

To investigate the expression profile of induced *svchs*, hairy root cultures were treated with MeJA (200 μmol/L) and then harvested after 6, 12, 24, 48 and 72 h. Total RNA was isolated from treated and non-treated hairy roots as already described. The expression profile of *svchs* was implemented by one-step RT-PCR reactions with 1.0 μg of total RNA from each sample, and semi-quantitative one-step RT-PCR was done according to the manufacturer's instructions (TaKaRa). All of the RNA templates were digested with RNase-free DNAse I and coding-sequence-specific PCR primer pairs (fexsvchs and rexsvchs) were synthesized (Table S1). PCR amplifications were done in a volume of 25 μ L using the following conditions: 30 min at 50 °C and 2 min at 94 °C followed by 25 cycles of 30 s at 94 °C, 30 s at 55 °C and 2 min at 72 °C, and a final extension of 10 min at 72 °C. The 18S rRNA gene was used as an internal control to ensure that equal amounts of total RNA were used in the reactions. This gene was amplified using a one-step RT-PCR reaction and specific primers (18Sf and 18Sr; Table S1) from conserved regions of plant 18S rRNA. The PCR products were separated on 1% agarose gels stained with Goldview and the gray density of the target bands was measured using Gel-Pro Analyzer 4.0 (Media Cybernetics, Bethesda, MD, USA).

## Results and Discussion

###  Cloning of the full-length cDNA of *Svchs*

**Figure 2:**
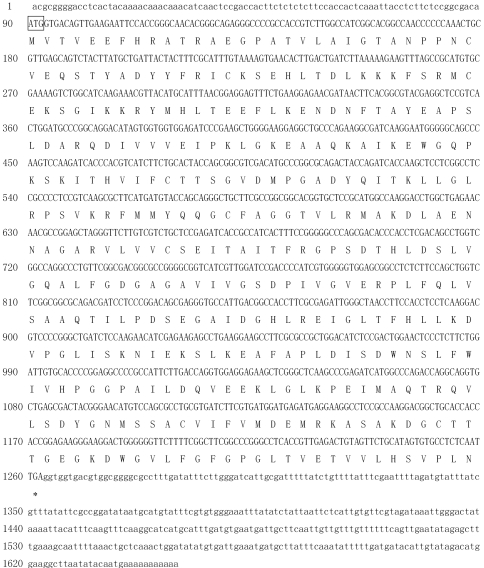
Full-length cDNA sequence and deduced amino acid sequence of SvCHS. The start (ATG) was boxed and the stop (TGA) codons was marked with asterisks, respectively. The coding sequence of *Svchs* is show in capital letters and the 5'- and 3'-untranslated regions are shown in normal letters.

Oligonucleotide primer pairs for amplification of the *S. viscidulachs* gene were designed based on the conserved regions of known *chs* genes from other plant specieses (*S. baicalensischs*-C, *S. baicalensischs*-P, *S. baicalensischs* and *Perilla frutescenschs*). A 597 bp cDNA fragment was amplified by RT-PCR and showed 95%, 82% and 82% sequence similarity with *chs* genes from *S. baicalensis*, *Torenia hybrida* and *Anthurium andraeanum*, respectively, indicating that conserved sequences of the putative *svchs* gene had been successfully isolated from *S. viscidula*. Gene-specific primers were subsequently designed based on the internal sequence of the core fragment and used to amplify 3'-end and 5'-end cDNA. A 3'-RACE product of ~531 bp and a 5'-RACE product of ~632 bp were isolated and sequenced. Finally, the full-length cDNA of *svchs* was deduced by aligning and assembling the sequences of the core fragment and the 5'-RACE and 3'-RACE ends, followed by confirmation via RT-PCR using a pair of additional full-length cDNA primers. Sequence analysis revealed that the cloned full-length cDNA of the *svchs* gene (GenBank accession no. EU386767) was 1649 bp long and contained a 1170 bp ORF that encoded a 390 amino acid protein; the gene was flanked by an 89 bp 5'-untranslated region (UTR) and a 390 bp 3'-UTR with a putative polyadenylation signal AATAA at a position 100 bp downstream from the stop codon (Figure 2).

###  Structural characterization and physicochemical properties

The protein coded by the *svchs* gene had a calculated molecular mass of 42.56 kDa and a theoretical isoelectric point of 5.79. A BLASTP comparison of the deduced amino acid sequence of SvCHS with that of other CHS proteins revealed high homology between SvCHS and the corresponding proteins of plants such as *Antirrhinum majus* (88% identity, 95% positivity), *Misopates orontium* (87% identity, 94% positivity), *Perilla frutescens* (87% identity, 94% positivity) and *S. baicalensis* (CHS: 97% identity, 98% positivity; CHS-P: 95% identity, 98% positivity; CHS-C: 94% identity, 97% positivity), indicating that SvCHS belonged to a CHS protein family. Analysis of the subcellular localization by using the TargetP 1.1 server suggested that SvCHS was restricted to the cytoplasm, a finding that agreed with the absence of a signal peptide in the protein and with the demonstration by Hrazdina (1992) that flavonoids are synthesized in the cytoplasm. In addition, the TMHMM Server v.2.0 and ProtScale predicted that SvCHS was a hydrophilic protein with no transmembrane structures; this observation implied that SvCHS catalyzes the biosynthesis of narigenin directly in the cytoplasm, without protein sorting.

**Figure 3:**
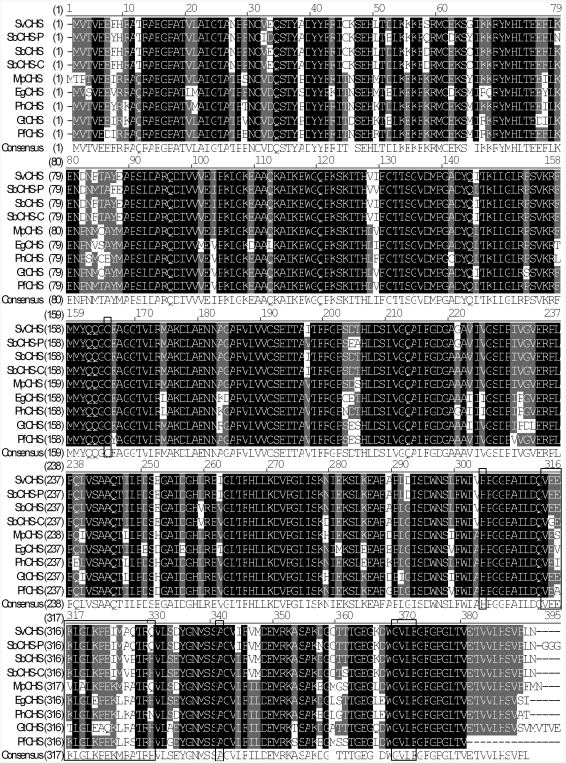
Multiple sequence alignment of SvCHS and other plant CHS proteins. Identical sites are shown in white letters on a black background, conserved sites are shown in white letters on a gray background and other sites are shown in black letters on a white background. The highly conserved catalytic domain motif Cys-His-Asn, an N-myristoylation motif gvlfgf and a malonyl-CoA binding motif veeklglkpeimaqtrq are boxed. The database accession numbers of the sequences used in the alignment are: *Eustoma grandiflorum* (EgCHS, GenPept accession no. AB078953), *Gentiana triflora* (GtCHS, GenPept accession no. D38043), *Mazus pumilus* (MpCHS, GenPept accession no. AY131328), *Perilla frutescens* (PfCHS, GenPept accession no. AB002582), *Petunia x hybrida* (PhCHS, GenPept accession no. X14591), *Scutellaria baicalensis* (SbCHS-C, GenPept accession no. AB046666), *Scutellaria baicalensis* (SbCHS-P, GenPept accession no. AF035622), *Scutellaria baicalensis* (SbCHS, GenPept accession no. AB008748) and *Scutellaria viscidula* (SvCHS, GenPept accession no. EU386767).

Full-length sequence alignment with Vector NTI 8.0 showed that SvCHS has three highly conserved residues (Cys_164_, His_304_ and Asn_340_; Figure 3) that form the active center of the CHS catalytic domains (Jez and Noel, 2000). PROSITE predicted that SvCHS contained a binding site (positions 313-330) for malonyl-CoA, a precursor that is cyclized to produce chalcone. In addition, a N-myristoylation site was found at positions 368-373, and myristoylated residues may bind to a hydrophobic core and stabilize the protein structure. The presence of these conserved amino acid residues in the aligned sequences suggested that SvCHS could catalyze the synthesis of chalcone in *S. viscidula*.

###  Phylogenetic analysis

**Figure 4:**
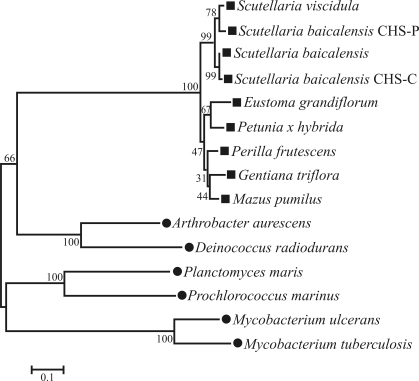
Phylogenetic analysis of plant and bacterial CHS. The phylogenetic tree was constructed by the Neighbor-Joining method (based on 1000 bootstrap replicates) using MEGA3 software; the bootstrap values are shown on the branches. Plant species are indicated by (■) and bacterial species by (•). The database accession numbers of the sequences used in the alignment are: *Arthrobacter aurescens* (AaCHS, GenPept accession no. NC_008711), *Deinococcus radiodurans* (DrCHS, GenPept accession no. NC_001264), *Mycobacterium tuberculosis* (MtCHS, GenPept accession no. CP000717), *Mycobacterium ulcerans* (MuCHS, GenPept accession no. NC_008611), *Planctomyces maris* (PmCHS, GenPept accession no. NZ_ABCE01000005) and *Prochlorococcus marinus* (PmCHS, GenPept accession no. NC_005071); the abbreviations and accession numbers for the other protein sequences are indicated in the legend for Figure 3.

*chs* gene sequences of a variety of plants and bacteria have been reported, with considerable attention being given to the genetic engineering of flavonoids. To examine the phylogenetic relationships among *chs* genes (Jiang and Cao, 2007), the *chs* genes of nine plants were aligned (Figure 4). As expected, *S. viscidula* was closely related to *S. baicalensis* since both belong to the same genus; the relationship observed with the remaining *chs* genes indicated that this gene encoded a highly conserved enzyme involved in flavonoid biosynthesis.

###  Three-dimensional model of SvCHS

**Figure 5:**
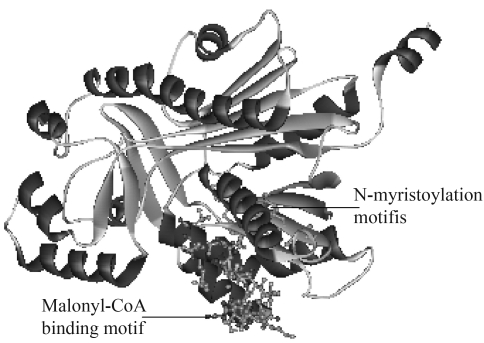
3D structural model of SvCHS. The α-helix and extended strand are indicated in red and blue, respectively. Random coils are indicated in silver. Selected important motifs are indicated.

Homology-based 3-D structural modeling of SvCHS was done using the program SWISS-MODEL based on the crystal structure of *Medicago sativa* CHS and subsequently displayed with WelLab ViewerLite. As shown in Figure 5, the secondary structure of SvCHS consisted of random coils, α -helixes and extended strands, with the former two being the main components (43.6% and 37.4%, respectively). Most of the recognized motifs occurred in the coiled-coil structure of SvCHS, in agreement with the fact that coiled-coils generally contain most of the significant motifs associated with biological functions in a variety of proteins. This analysis indicated that SvCHS had the typical molecular structure of CHS in general and compared favorably with the experimental data for *M. sativa* CHS (Ferrer *et al.*, 1999; Jez and Noel, 2000).

###  Expression profile of *Svchs* following exposure to MeJA

**Figure 6:**
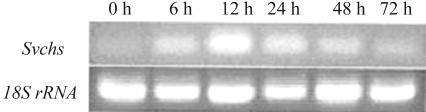
Expression profile of *Svchs* following exposure to MeJA for different lengths of time.  Upper panel – *Svchs* expression, lower panel – 18S rRNA levels used to normalize the amount of template used in the PCR reactions.

Short-term exposure to MeJA increases the production of the flavonoid baicalin, whereas long-term exposure inhibits plant cell growth (Zhang and Xu, 2006). We used a one-step RT-PCR with the primers fexsvchs and rexsvchs to investigate the changes in *svchs* expression following exposure to MeJA. Treatment with MeJA stimulated *svchs* expression, with the greatest increase occurring after a 12 h exposure, followed by a progressive decrease in the level of stimulation (Figure 6). This finding indicated that *svchs* expression was sensitive to MeJA and that the levels of SvCHS may be modulated by this phytohormone. The enhanced expression of *svchs* in the presence of MeJA would be expected to result in greater CHS activity and the accumulation of plant secondary metabolites in *S. viscidula*, a conclusion in agreement with the observations of Sánchez-Sampedro *et al.* (2005) that MeJA enhanced CHS activity and increased the levels of silymarin in cell cultures of *Silybum marianum*.

In summary, we have cloned and characterized an *svchs* gene from the deciduous medicinal herb *S. viscidula*. The protein encoded by this gene (SvCHS) shares various structural similarities with the corresponding proteins from other plants, especially *S. baicalensis*, including a binding site for malonyl-CoA and an N-myristoylation motif. These structural similarities suggest that SvCHS may be involved in plant intermediate metabolism. Semi-quantitative one-step RT-PCR showed that *svchs* was responsive to MeJA at the transcriptional level, and this could provide an important mechanism for enhancing intracellular flavonoid levels. These results provided useful information on the induction and regulation of genes involved in flavonoid biosynthesis. Based on the cloning and characterization of this gene, we have constructed a plant expression vector containing *svchs* that is currently being used to genetically transform *S. viscidula* in order to assess the role of CHS in flavonoid production.

## Supplementary Material

The following online material is available for this article:

Table S1 Primers used in molecular cloning and expression profiling of the Svchs gene.

This material is made available as part of the online article available from http://www.scielo.br.gmb.
