# Supplementary material for: Molecular cloning and expression profiling of a chalcone synthase gene from hairy root cultures of Scutellaria viscidula Bunge
Source: Genet Mol Biol. 2010 Jun 1;33(2):285–91. doi: 10.1590/S1415-47572010005000031 (PMC3036846; doi:10.1590/S1415-47572010005000031)
Supplement: Table S1 — Primers used in molecular cloning and expression profiling of the Svchs gene. [file gmb-33-2-285-suppl1.pdf]

Table S1 - Primers used in molecular cloning and expression profiling of the *Svchs* gene.

Primers	Sequence
dfchs	5'-TTCATGATGTACCAGCAGGGCTGCT-3'
drchs	5'-GGAGG(A/C)CTTCCTCATCTCATCCA-3'
svchs3-1	5'-TAACCTTCCACCTCCTCAAGGAC-3'
svchs3-2	5'-TTCGTGATGGATGAGATGAGGAAG-3'
svchs5-1	5'-CGTCAATGGCACCCTCGCTGTC-3'
svchs5-2	5'-TGATGGCGGTGATCTCGGAGCAGA-3'
fsvchs	5'-ACGCGGGGACCTCACTACAAAA-3'
rsvchs	5'-CATTGTATATTAAGCCTTCCATG-3'
fexsvchs	5'-ATGGTGACAGTTGAAGAATTCCA-3'
rexsvchs	5'-ATTGAGAGGCACACTATGCAGAA-3'
18Sf	5'-ATGATAACTCGACGGATCGC-3'
18Sr	5'-CTTGGATGTGGTAGCCGTTT-3'
UPM	Long: 5'-CTAATACGACTCACTATAGGGCAAGCAGTGGTATCAACGCAGAGT-3'<br>Short: 5'-CTAATACGACTCACTATAGGGC-3'
NUP	5'-AAGCAGTGGTATCAACGCAGAGT-3'

dfchs – deoxyribonucleotide forward chs, drchs – deoxyribonucleotide reverse chs, svchs – *Scutellaria viscidula* chs, 3-1, 3-2, 5-1 and 5-2 [3 and 5 refer to 3'end and 5'end, respectively; 1 and 2 refer to the first and the second, respectively], fsvchs – forward svchs, fexsvchs – forward expression svchs, rsvchs – reverse svchs, rexsvchs – reverse expression svchs, NUP – nested universal primer, UPM – universal primer mix, and 18Sf and 18Sr – 18 s rRNA forward (f) and reverse (r) primers, respectively.
